# Supplementary material for: Optimal Designs for Identifying Effective Doses in Drug Combination Studies
Source: Biom J. 2026 Sep 28;68(5):e70186. doi: 10.1002/bimj.70186 (PMC13620475; doi:10.1002/bimj.70186)
Supplement: Supplementary file 1 [file BIMJ-68-e70186-s001.pdf]

# Supplementary Material: Proof of Theorem 3.2 in "Optimal designs for identifying effective doses in drug combination studies"

Leonie Schürmeyer[1], Ludger Sandig[1], Jorrit Kühne[1], Leonie T. Hezler[2], Bernd-Wolfgang Igl[2], and Kristen Schorning[1]

[1]Department of Statistics, TU Dortmund University, Vogelpothsweg 87, Dortmund, 44227, North Rhine-Westphalia, Germany
[2]Global Experimental Medicine, Data and Analytics, Boehringer Ingelheim Pharma GmbH & Co. KG, Birkendorferstraße 65, Biberach an der Riß, 88397, Baden-Wuerttemberg, Germany

This document provides supplementary material to the paper "Optimal designs for identifying effective doses in drug combination studies". In particular, we present a detailed proof of Theorem 3.2.

We follow the principal idea of the proof of [1]. First, we consider the case where $M(\xi^*, \theta)$ is non-singular. Consequently, it holds that $\frac{\partial}{\partial\theta}\eta((c,d),\theta) \in \text{range}(M(\xi^*,\theta))$. Let

$$\mathcal{M} = \{M(\xi,\theta) \mid \xi \text{ is a design on } \mathcal{Z}\}$$

be the set of information matrices, where $M(\xi,\theta)$ is defined in (5). Note that $\mathcal{M}$ is a subset of all non-negative definite $(p \times p)$-matrices. For a combination $(c,d) \in \mathcal{Z}$, we introduce the function

$$\tilde{\varphi}(\,\cdot\,,(c,d)) : \mathcal{M} \to [0,\infty), \quad \tilde{\varphi}(M,(c,d)) = \frac{\partial}{\partial\theta}\eta((c,d),\theta)^T M^- \frac{\partial}{\partial\theta}\eta((c,d),\theta), \tag{1}$$

and the function

$$\tilde{\Phi}_{\text{MED}_q} : \mathcal{M} \to [0,\infty), \quad \tilde{\phi}_{\text{MED}_q}(M) = \left(\int_{\mathcal{C}(\theta)} \left(\tilde{\varphi}(M,(c,d))\right)^q d\mu((c,d))\right)^{\frac{1}{q}}. \tag{2}$$

Note that the functions in (1) and (2) are obtained by adapting the functions defined in (4) and (10). Because of the convexity of $\phi_{\text{MED}_q}$ (with respect to $\xi$) and the convexity of the set $\mathcal{M}$, the function $\tilde{\phi}_{\text{MED}_q}$ is also a convex function. Moreover, a design is $\xi^*$ is locally $\text{MED}_q$-optimal if and only if the corresponding information matrix $M^* = M(\xi^*,\theta)$ minimizes the function $\tilde{\phi}$ on the set $\mathcal{M}$. Because of the convexity of $\tilde{\phi}_{\text{MED}_q}$, the design $\xi^*$ is locally $\text{MED}_q$-optimal if and only if the directional derivative of $\tilde{\phi}_{\text{MED}_q}$ evaluated in $M^*$ is nonnegative for all directions $E_0 = E - M^*$, where $E \in \mathcal{M}$. Given that $\mathcal{M}$ is the convex hull of the set

$$\mathcal{D} = \left\{M(\delta_{(c_0,d_0)},\theta) \mid (c_0,d_0) \in \mathcal{Z}\right\},$$

where $\delta_{(c_0,d_0)}$ is the Dirac measure at the combination $(c_0,d_0)$, it is sufficient to prove the inequality for all $E \in \mathcal{D}$.

We now provide a step-by-step proof where we calculate the directional derivative $\partial\tilde{\phi}_{\mathrm{MED}_q}(M^*, E_0)$ of $\tilde{\phi}_{\mathrm{MED}_q}(M^*)$ in direction $E_0 \in \mathcal{M}$. If $q \geq 1$, the $L_q(\mu)$-norm (of a non-negative function) is differentiable. Combined with the chain rule, we obtain

$$\partial\tilde{\phi}_{\mathrm{MED}_q}(M^*, E_0) = \left(\int_{\mathcal{C}(\theta)} \left(\tilde{\varphi}(M^*, (c,d))\right)^q d\mu((c,d))\right)^{\frac{1}{q}-1} \cdot \int_{\mathcal{C}(\theta)} \left(\tilde{\varphi}(M^*, (c,d))\right)^{q-1} \partial\tilde{\varphi}\big(M^*, E_0, (c,d)\big)\, d\mu((c,d)), \quad (3)$$

where $\partial\tilde{\varphi}(M^*, E_0, (c,d))$ denotes the directional derivative of $\tilde{\varphi}(M^*, (c,d))$ in direction $E_0$ for a specific combination $(c,d) \in \mathcal{C} \subset \mathcal{Z}$. The next step depends on whether the information matrix is singular.

1. If the matrix $M^* = M(\xi^*, \theta)$ is non-singular, the function $\tilde{\varphi}(\,\cdot\,, (c,d))$ is differentiable in $M^*$ and the directional derivative $\partial\tilde{\varphi}(M^*, E_0, (c,d))$ is well-defined. To calculate $\partial\tilde{\varphi}(M^*, E_0, (c,d))$, we follow the definition in [4]

$$\begin{aligned}
\partial\tilde{\varphi}(M^*, E_0, (c,d)) &= \lim_{\varepsilon\to 0} \frac{1}{\varepsilon}\big(\tilde{\varphi}(M^* + \varepsilon E_0) - \tilde{\varphi}(M^*)\big) \\
&= \lim_{\varepsilon\to 0} \frac{1}{\varepsilon} \frac{\partial}{\partial\theta}\eta((c,d),\theta)^T \left((M^* + \varepsilon E_0)^{-1} - (M^*)^{-1}\right) \frac{\partial}{\partial\theta}\eta((c,d),\theta) \\
&= g'(0)
\end{aligned}$$

for $g(\varepsilon) := \frac{\partial}{\partial\theta}\eta((c,d),\theta)^T (M^* + \varepsilon E_0)^{-1} \frac{\partial}{\partial\theta}\eta((c,d),\theta)$. With the rules from matrix differential calculus [2, pp. 148, 151, 176] we compute

$$\begin{aligned}
\mathrm{d}[g] &= \frac{\partial}{\partial\theta}\eta((c,d),\theta)^T\, \mathrm{d}\big[(M^* + \varepsilon E_0)^{-1}\big] \frac{\partial}{\partial\theta}\eta((c,d),\theta) \\
&= -\frac{\partial}{\partial\theta}\eta((c,d),\theta)^T (M^* + \varepsilon E_0)^{-1}\, \mathrm{d}\big[M^* + \varepsilon E_0\big] (M^* + \varepsilon E_0)^{-1} \frac{\partial}{\partial\theta}\eta((c,d),\theta) \\
&= -\frac{\partial}{\partial\theta}\eta((c,d),\theta)^T (M^* + \varepsilon E_0)^{-1} E_0 (M^* + \varepsilon E_0)^{-1} \frac{\partial}{\partial\theta}\eta((c,d),\theta)\, \mathrm{d}[\varepsilon],
\end{aligned}$$

hence we have

$$g'(0) = -\frac{\partial}{\partial\theta}\eta((c,d),\theta)^T (M^*)^{-1} E_0 (M^*)^{-1} \frac{\partial}{\partial\theta}\eta((c,d),\theta).$$

We continue with the original derivative,

$$\begin{aligned}
\partial\tilde{\varphi}((M^*, E_0, (c,d)) &= \frac{\partial}{\partial\theta}\eta((c,d),\theta)^T \left(-(M^*)^{-1} E_0 (M^*)^{-1}\right) \frac{\partial}{\partial\theta}\eta((c,d),\theta) \\
&= \frac{\partial}{\partial\theta}\eta((c,d),\theta)^T \left(-(M^*)^{-1} (M^* - E)(M^*)^{-1}\right) \frac{\partial}{\partial\theta}\eta((c,d),\theta) \\
&= \frac{\partial}{\partial\theta}\eta((c,d),\theta)^T \left((M^*)^{-1} E (M^*)^{-1}\right) \frac{\partial}{\partial\theta}\eta((c,d),\theta) \\
&\quad - \frac{\partial}{\partial\theta}\eta((c,d),\theta)^T (M^*)^{-1} \frac{\partial}{\partial\theta}\eta((c,d),\theta)\,.
\end{aligned}$$

If $E_0 = E - M^*$, where $E = M(\delta_{c_0,d_0}, \theta) \in \mathcal{D}$, the partial derivative further reduces to

$$\partial\tilde{\varphi}((M^*, E_0, (c,d)) = \tilde{\alpha}^2\big((c_0,d_0), (c,d), M^*\big) - \tilde{\varphi}(M, (c,d)), \quad (4)$$

where the function $\tilde{\alpha}$ is given by

$$\tilde{\alpha}\big((c_0,d_0), (c,d), M, \theta\big) = \frac{\partial}{\partial\theta}\eta((c,d),\theta)^T M^{-1} \frac{\partial}{\partial\theta}\eta((c_0,d_0),\theta).$$

Using (3) in combination with (4), we obtain:

$$
\begin{aligned}
&\partial\tilde{\phi}_{\mathrm{MED}_q}(M^*, E_0)\\
&= \left(\int_{\mathcal{C}(\theta)} \big(\tilde{\varphi}(M^*,(c,d))\big)^q d\mu((c,d))\right)^{\frac{1}{q}-1} \int_{\mathcal{C}(\theta)} \big(\tilde{\varphi}(M^*,(c,d))\big)^{q-1} \tilde{\varphi}(M^*,(c,d))\, d\mu((c,d))\\
&\quad - \left(\int_{\mathcal{C}(\theta)} \big(\tilde{\varphi}(M^*,(c,d))\big)^q\, d\mu((c,d))\right)^{\frac{1}{q}-1}\\
&\quad \cdot \int_{\mathcal{C}(\theta)} \big(\tilde{\varphi}(M^*,(c,d))\big)^{q-1} \tilde{\alpha}^2\big(M^*,(c_0,d_0),(c,d)\big)\, d\mu((c,d))\\
&= \left(\int_{\mathcal{C}(\theta)} \big(\tilde{\varphi}(M^*,(c,d))\big)^q\, d\mu((c,d))\right)^{\frac{1}{q}}\\
&\quad - \left(\int_{\mathcal{C}(\theta)} \big(\tilde{\varphi}(M^*,(c,d))\big)^q\, d\mu((c,d))\right)^{\frac{1}{q}} \left(\int_{\mathcal{C}(\theta)} \tilde{\varphi}(M^*,(c,d))\, d\mu((c,d))\right)^{-1}\\
&\quad \cdot \int_{\mathcal{C}(\theta)} \big(\tilde{\varphi}(M^*,(c,d),\theta)\big)^{q-1} \tilde{\alpha}^2\big((c_0,d_0),(c,d),M^*,\theta\big)\, d\mu((c,d))\\
&= \tilde{\phi}_{\mathrm{MED}_q}(M^*) \left[1 - \big(\tilde{\phi}_{\mathrm{MED}_q}(M^*)\big)^{-q} \int_{\mathcal{C}(\theta)} \beta\big((c_0,d_0),(c,d),M^*\big)\, d\mu(c,d)\right],
\end{aligned}
$$

where the function $\beta$ is given by

$$
\beta\big((c_0,d_0),(c,d),M\big) = \big(\tilde{\varphi}(M,(c,d),\theta)\big)^{q-1} \tilde{\alpha}^2\big((c_0,d_0),(c,d),M,\theta\big).
$$

Consequently, a design $\xi^*$ is $\mathrm{MED}_q$-optimal if and only if for the corresponding information matrix $M(\xi^*,\theta)$ the inequality

$$
\int_{\mathcal{C}(\theta)} \beta\big((c_0,d_0),(c,d),M^*\big)\, d\mu(c,d) - \big(\tilde{\phi}_{\mathrm{MED}_q}(M^*)\big)^q \leq 0.
$$

holds for all $(c_0,d_0) \in \mathcal{Z}$ or in terms of the design if and only if the inequality

$$
\int_{\mathcal{C}(\theta)} \big(\varphi((c,d),\xi^*,\theta)\big)^{q-1} \alpha^2\big((c_0,d_0),(c,d),\xi^*,\theta\big)\, d\mu(c,d) - \big(\phi_{\mathrm{MED}_q}(\xi^*,\theta)\big)^q \leq 0 \tag{5}
$$

holds for all $(c_0,d_0) \in \mathcal{Z}$. This proves the first part of the first assertion. It needs to be proven that equality holds for any support point $(c_0,d_0) \in \mathrm{supp}(\xi^*)$. Therefore, we assume the opposite, that there is strict inequality in (5). This leads to

$$
\int_{\mathcal{Z}} \int_{\mathcal{C}(\theta)} \big(\varphi((c,d),\xi^*,\theta)\big)^{q-1} \alpha^2\big((c_0,d_0),(c,d),\xi^*,\theta\big)\, d\mu(c,d)\, d\xi((c_0,d_0)) < \big(\phi_{\mathrm{MED}_q}(\xi^*,\theta)\big)^q. \tag{6}
$$

Using that

$$
\begin{aligned}
&\int_{\mathcal{Z}} \alpha^2\big((c_0,d_0),(c,d),\xi^*,\theta\big)\, d\xi((c_0,d_0))\\
&= (\eta((c,d),\theta))^T M^{-1}(\xi,\theta)\eta((c_0,d_0),\theta)(\eta((c_0,d_0),\theta))^T M^{-1}(\xi,\theta)\eta((c,d),\theta)\ d\xi((c_0,d_0))\\
&= (\eta((c,d),\theta))^T M^{-1}(\xi,\theta) M(\xi,\theta) M^{-1}(\xi,\theta)\eta((c,d),\theta)\\
&= (\eta((c,d),\theta))^T M^{-1}(\xi,\theta)\eta((c,d),\theta),
\end{aligned}
$$

the left-hand side of inequality (6) can be rewritten by:

$$\int_{\mathcal{Z}} \int_{\mathcal{C}(\theta)} \big(\varphi((c,d),\xi^*,\theta)\big)^{q-1} \alpha^2\big((c_0,d_0),(c,d),\xi^*,\theta\big)\, d\mu(c,d)\, d\xi((c_0,d_0)) = \big(\phi_{\mathrm{MED}_q}(\xi^*,\theta)\big)^q.$$

Consequently, equality must hold in (5) for $(c_0,d_0) \in \mathrm{supp}(\xi^*)$.

2. If $M^* = M(\xi^*,\theta)$ is singular and satisfies the range inclusion

$$\frac{\partial}{\partial\theta}\eta((c,d),\theta) \in \mathrm{range}(M(\xi^*,\theta)) \quad \text{for all } (c,d) \in \mathcal{C}(\theta),$$

the function $\tilde{\varphi}(\,\cdot\,,(c_0,d_0))$ is not differentiable in $M^*$ resulting in the non-differentiability of $\tilde{\phi}_{\mathrm{MED}_q}$. However, in this case, a similar result like in the first part of the theorem is obtained using the concept of convex analysis and subgradients. More precisely, the matrix $M^*$ minimizes the function $\tilde{\phi}_{\mathrm{MED}_q}$ over all $M \in \mathcal{M}$ if and only if there exists a subgradient $N$ of $\tilde{\phi}_{\mathrm{MED}_q}$ such that

$$\mathrm{tr}[NE_0] \geq 0 \tag{7}$$

for all directions $E_0 = E - M^*$, where $E \in \mathcal{M}$. Following [3, p. 170], the set of all subgradients of the function $\tilde{\varphi}(\,\cdot\,,(c_0,d_0))$ in $M^*$ is given by

$$D\tilde{\varphi}(M_0,(c,d)) = \left\{ \frac{\partial}{\partial\theta}\eta((c,d),\theta) G \frac{\partial}{\partial\theta}\eta((c,d),\theta)^T G^T \;\middle|\; G \in (M^*)^- \right\},$$

where $(M^*)^-$ is the set of all generalized inverses of $M^*$. Due to the differentiability of the $L_q$-norm the set of all subgradients of $\tilde{\phi}_{\mathrm{MED}_q}$ in $M^*$ is given by

$$\begin{aligned} D\tilde{\phi}_{\mathrm{MED}_q}(M^*) = \Bigg\{ & \left( \int_{\mathcal{C}(\theta)} \big(\tilde{\varphi}(M^*,(c,d))\big)^q \, d\mu((c,d)) \right)^{\frac{1}{q}-1} \\ & \cdot \int_{\mathcal{C}(\theta)} \big(\tilde{\varphi}(M^*,(c,d))\big)^{q-1} N \, d\mu((c,d)) \;\Bigg|\; N \in D\tilde{\varphi}(M^*,(c,d)) \Bigg\}. \end{aligned} \tag{8}$$

The first assertion of the second part of the theorem follows by using (7) with (8). The proof of the second assertion is analogous to the proof given in the first part. □

## References


[1] Holger Dette and Kirsten Schorning. Optimal designs for comparing curves. *Annals of Statistics*, 44(3):1103, 2016.

[2] Jan R. Magnus and Heinz Neudecker. *Matrix Differential Calculus with Applications in Statistics and Econometrics*. John Wiley & Sons Ltd, Chichester, 2nd edition, 1999.

[3] Friedrich Pukelsheim. *Optimal Design of Experiments*. SIAM, Philadelphia, 2006.

[4] Samuel Silvey. *Optimal Design. An Introduction to the Theory for Parameter Estimation*, volume 1. Springer Science & Business Media, 2013.
